# Seed priming with gas plasma-activated water in Ethiopia’s “orphan” crop tef (*Eragrostis tef*)

**DOI:** 10.1007/s00425-024-04359-5

**Published:** 2024-02-26

**Authors:** Lena M. M. Fatelnig, Solomon Chanyalew, Mahilet Tadesse, Worku Kebede, Nigusu Hussein, Felipe Iza, Zerihun Tadele, Gerhard Leubner-Metzger, Tina Steinbrecher

**Affiliations:** 1 Department of Biological Sciences, Royal Holloway University of London, Egham, Surrey TW20 0EX UK; 2 Debre Zeit Agricultural Research Center, Ethiopian Institute of Agricultural Research, P.O. Box 32, Debre Zeit, Ethiopia; 3 Electrical and Manufacturing Engineering, Wolfson School of Mechanical, Loughborough University, Leicestershire, LE11 3TU UK; 4 Division of Advanced Nuclear Engineering, Pohang University of Science and Technology (POSTECH), Pohang, Gyeongbuk 790-784 South Korea; 5 Institute of Plant Sciences, University of Bern, Altenbergrain 21, CH-3013 Bern, Switzerland; 6 Laboratory of Growth Regulators, Institute of Experimental Botany, Palacký University, Czech Academy of Sciences, Olomouc, Czech Republic

**Keywords:** Accelerated ageing, Gas plasma priming, Plasma agriculture, Seed germination, Seed technology

## Abstract

**Main conclusion:**

Seed priming with gas plasma-activated water results in an increased ageing resilience in *Eragrostis tef* grains compared to a conventional hydropriming protocol.

**Abstract:**

Tef (*Eragrostis tef*) is a cereal grass and a major staple crop of Ethiopia and Eritrea. Despite its significant importance in terms of production, consumption, and cash crop value, tef has been understudied and its productivity is low. In this study, tef grains have undergone different priming treatments to enhance seed vigour and seedling performance. A conventional hydropriming and a novel additive priming technology with gas plasma-activated water (GPAW) have been used and tef grains were then subjected to germination performance assays and accelerated ageing. Tef priming increases the germination speed and vigour of the grains. Priming with GPAW retained the seed storage potential after ageing, therefore, presenting an innovative environmental-friendly seed technology with the prospect to address variable weather conditions and ultimately food insecurity. Seed technology opens new possibilities to increase productivity of tef crop farming to achieve a secure and resilient tef food system and economic growth in Ethiopia by sustainable intensification of agriculture beyond breeding.

**Supplementary Information:**

The online version contains supplementary material available at 10.1007/s00425-024-04359-5.

## Introduction

Tef is a cereal grass and staple crop of Ethiopia, mainly cultivated for human consumption but also as fodder for livestock (Tadele 2019). Tef has several advantages over other crops, it can adapt to a wide range of environments such as moisture stress, poorly drained soils, and different altitudes. It is also less prone to diseases and pests compared to maize, wheat, and barley (Cannarozzi et al. 2014; Girija et al. 2021). Despite its significant importance in terms of production, consumption, and cash crop value, tef has long been neglected and its productivity is relatively low (Tadele 2019).

Seed priming is a pre-sowing treatment often applied to commercial seed lots and is widely used by seed technologists to enhance seed vigour and seedling performance (Corbineau et al. 2023). It is a technique that allows the controlled hydration of a seed and triggers metabolic processes of the early phase of germination without leading to full germination (radicle protrusion). The treatment provides faster and synchronized germination and an increased stress tolerance (Paparella et al. 2015; Ibrahim 2016). In general, storing seeds for long periods of time causes their deterioration and a main limitation of current priming techniques is increased susceptibility to ageing (reduced seed storability) (Fabrissin et al. 2021).

The global demand for food is rising and new sustainable and ecological approaches in agriculture are being adopted. Vertical farming, hydroponics, microorganisms, genetic engineering and also gas plasma agriculture are among those techniques. Gas plasma has recently received considerable attention for its application in sterilization and agriculture for its potential to enhance seed germination and healthy plant development (Bourke et al. 2018; Weltmann et al. 2019; Masiello et al. 2021; Waskow et al. 2021). Gas plasma is often called “the fourth state of matter,” along with solid, liquid and gas. Low temperature plasmas are ionised gases containing cocktails of electrons, ions, radicals, excited species, and photons under conditions that are far from thermodynamic equilibrium. Reactive species including free radicals, reactive oxygen species (ROS) and reactive nitrogen species (RNS) are produced at ambient temperature and atmospheric pressure and have applications in biology and medicine (Graves 2012; Grainge et al. 2022b). Gas plasma-activated water (GPAW) is produced by exposing water to a gas plasma that is sustained by applying high voltage electrical energy to a background gas. This results in the ionisation of the gas and the synthesis of a myriad of ions, reactive species, and free electrons (Wright et al. 2018; Zhou et al. 2020). GPAW has been shown to affect seed germination (Bafoil et al. 2018) and to break seed dormancy (Grainge et al. 2022a, b).

In this study, we use GPAW and classic hydropriming to treat different accessions of tef to compare germination, artificial ageing as well as seedling performance in the glasshouse and field. The ultimate goal of applying novel seed technologies to orphan crops is to increase the productivity of crop farming which will achieve a secure and resilient tef food system and economic growth in Ethiopia by sustainable intensification.

## Materials and methods

### Study species

Four tef [(*Eragrostis tef* (Zucc.) Trotter] germplasm were provided by the University of Bern (Switzerland), and additionally two commercial seed lots were used in this study. From the University of Bern, three white seed lots of the Tsedey tef variety harvested in different years and seasons, and one brown tef variety were used. The commercial tef seeds bought from Lovegrass (Kenly, UK) contained one white (batch no 20120) and one brown seed lot (batch no 200707). Tef grains used for this study did not have dormancy.

### Imaging

Grains were imaged using a light microscope (Leica MZ 125) and scanning electron microscopy (SEM) was conducted using a Hitachi S-3000N at an acceleration voltage of 20 kV. For the SEM, dry grains were mounted on 12.5 mm aluminium specimen stubs and carbon coated. X-Ray imaging was conducted using a COMPAI TrueView 100Pro at 50 kVp and 20 mAs (RPS Service Ltd., Byfleet, Surrey, UK).

### Germination in variable temperatures

As a standard germination assay, a triplicate set of grains were plated in Petri dishes (60 mm × 15 mm) with one sheet of filter paper (Macherey–Nagel 713, Düren, Germany) and 1 ml of deionised H_2_O. Three replicates with 50 tef grains each were used per temperature. The plates were incubated in the indicated temperature and radicle emergence was scored twice daily. To assess optimal and suboptimal temperature for germination of each seed lot, a germination assay was conducted in different temperatures between 12 and 32 °C using incubators (Panasonic MLR-352, Bracknell, UK).

### Priming methods

The initial moisture content of the tef grains was first determined by comparison of their fresh and dry weight using a Moisture analyser (HB43-S Mettler Toledo). Each seed batch was primed with a range of priming treatments (varying in length and target moisture content) to determine optimal priming conditions (in order to prevent under- or overpriming). Five hundred milligrams of grains were placed in the priming drums (4 cm diameter; 60 ml volume) with the required water volume achieving 20% target moisture content (MC per dry weight). The drums were constantly shaken at a speed of 8 rpm on the roller machine (Stuart roller mixer SRT9D) for 15 h at 21 °C. At the end of the treatment, the seeds were weighed and dried for 24 h at 24 °C. After drying back to the original moisture content, primed seeds were then subjected to a germination assay, and later to artificial ageing treatments. The plasma reactor engineered to produce GPAW consists of 12 high voltage AC electrodes covered in a dielectric material fixed below a gas permeable stainless-steel membrane (Wright et al. 2018; Grainge et al. 2022b). Air was used as a carrier gas (UN1002, BOC Ltd, Guildford, UK) which was led through the gas permeable membrane at a flow rate of 1 standard litre per minute (slm) into a chamber filled with 100 ml of deionised water. The plasma formed between the electrodes and the membrane produces reactive oxygen species (ROS) and reactive nitrogen species (RNS) that dissolve in the liquid creating the plasma activated water. The plasma was sustained at 15.8 kV and 27.1 kHz and it was modulated with an on-time of 100 ms and a duty cycle of 30%. The flow rate was controlled through an Alicat MC-series mass flow controller (Alicat Scientific, Tucson, AZ, USA) and the applied voltage was measured using a Tektronix P6015A high voltage probe (Tektronix, Beaverton, OR, USA) and a TBS1102B digital oscilloscope (Tektronix). Over 45 min, this generated the GPAW used for the treatment. The same priming moisture content and duration as in the hydropriming treatment were used (20% MC; 15 h).

### Artificial ageing

Chambers with a relative humidity (RH) of 70 and 80% were prepared using a defined concentration of lithium chloride (LiCl) solution (Hay et al. 2008). The triplicates of seeds (50 grains) were placed in Eppendorf tubes which were resting on a rack inside of a LiCl filled Tupperware box, and incubated in the three different relative humidities (60% RH, 70% RH, 80% RH) for 3 or 7 days at 42 °C. After completion of ageing treatment, seeds were subjected to a germination assay at different temperatures (from 12 to 20 °C). After no more new germinations occurred, non-germinated seeds were transferred into a new Petri dish with 100 µM GA_4+7_ (Duchefa Biochemie B.V, Haarlem, The Netherlands) and germination was further monitored for 1 week, to assess whether seeds were viable.

### Conductivity measurement

Three replicates of Petri dishes (5 cm) with 3 ml of water and 50 grains were placed on a shaker and incubated for 5 min to 7 h (5 min, 30 min, 1 h, 2 h, 3 h, 6 h, 7 h). One ml of the seed soak water was pipetted into 2 Eppendorf tubes per Petri dish. After letting the tubes equilibrate on the lab bench for at least 30 min, the conductivity of the seed soak water was measured with a JENWAY conductivity meter model 4510 (Cole-Palmer, Stone, UK). Each Eppendorf tube was measured twice to produce a technical replicate.

### Pot and field experiments

Hydroprimed, GPAW primed and untreated commercial tef grains (Lovegrass Ltd, Kenly, UK, white batch no 20120, brown batch no 200707) were planted in June 2021 in Ethiopia, Debre Zeit. Both priming treatments were conducted at 20% MC for 15 h. For the pot experiments, 50 grains per pot were planted per treatment (control, hydroprimed, GPAW primed) in three replicates. Seedling emergence was counted from day 3 to 6. For the biomass data, ten individual plants were randomly selected from each pot. Fresh weight and dry weight (after 3 days at 75 °C) was measured. For the field experiment, 0.4 g of grain was used per 1 m^2^. Grains were hand drilled in rows. Plant height, shoot biomass per plot and peduncle length (average length of the last apical internode of the main shoot culm) were measured.

### Data analysis

Germination percentage was calculated using the number of seeds that germinated in each Petri dish and the total amount of seeds that this dish contained. The germination percentage of primed and aged seeds was then plotted with a control using GraphPad for comparison of vigour and viability. Experiments were conducted in a randomized design and conducted in biological replicates as indicated in the respective method sections. Statistical differences between groups under comparison were determined by ANOVA followed by the appropriate post hoc tests (*P* = 0.05) using GraphPad PRISM v9 software.

## Results

### Grain morphology

**Fig. 1:**
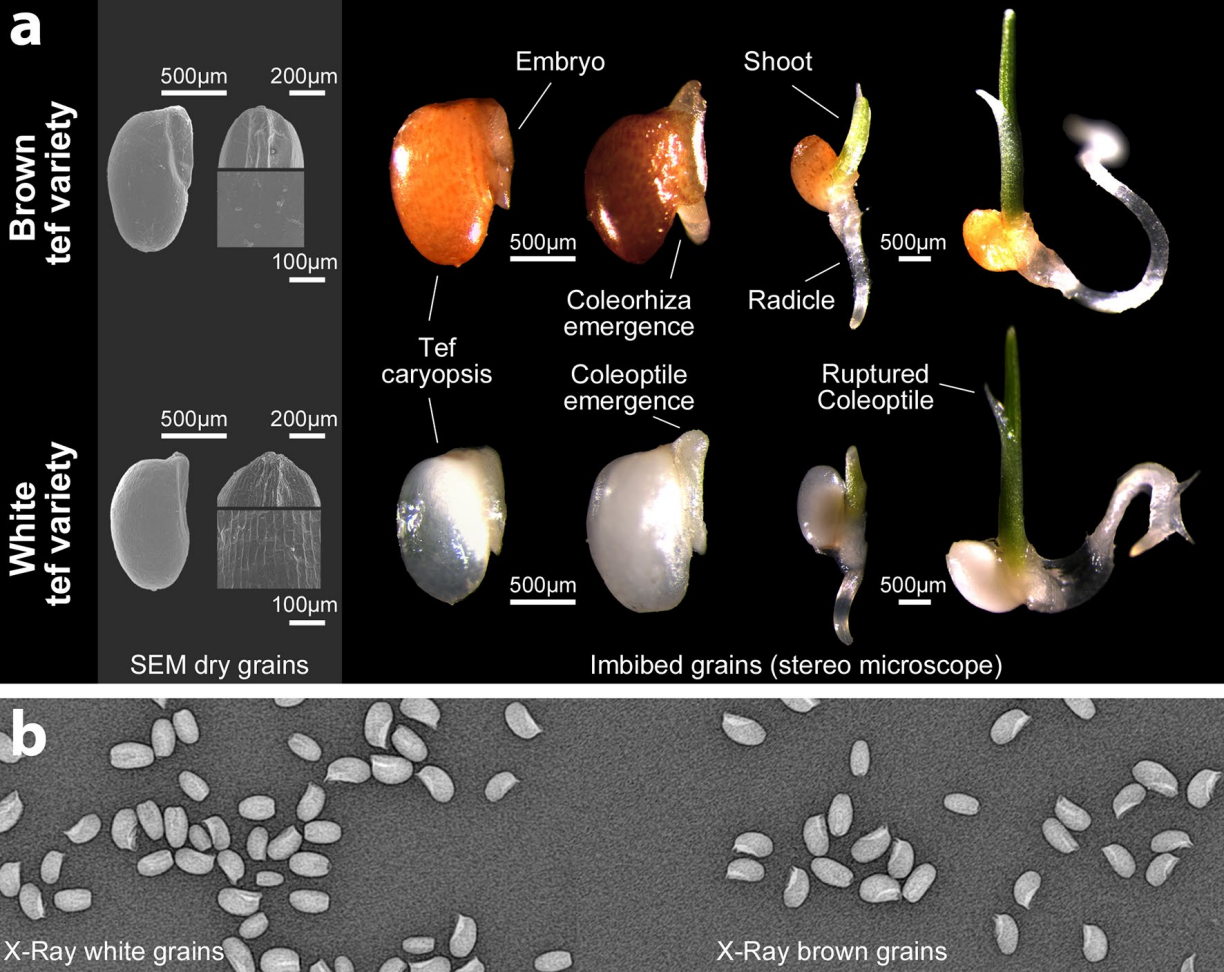
Morphology of white and brown tef grains. **a** Dry tef grains under a scanning electron microscope (SEM) show a typical caryopsis with the embryo on one side of the grain. A close-up shows the cell structure of the fruit coat. Imbibed grains under a stereo microscope reveal the typical phases of grain germination. During imbibition, the grain swells and coleoptile and coleorhiza became more pronounced until they rupture, and the shoot and radicle emerge. **b** X-ray of white and brown tef grains. In both varieties, the grain (caryopsis) is a single seeded fruit and the tef embryo is located in the dorsal region of the grain

Tef [*Eragrostis tef* (Zucc.) Trotter] belongs to the family of Poaceae, subfamily Eragrostoidae and its fruits constitute an oval-shaped caryopsis that shows a range of colours (from white to dark brown) (Fig. 1). After imbibition the coleoptile (sheath protecting the young shoot tip) and coleorhiza (sheath protecting the young root) start to emerge and subsequently get ruptured by the shoot and the radicle. The germination of the diaspore is completed by visible radicle emergence on the distal end of the grain. The seed coat of the white grain varieties shows a pronounced regular cell structure, particularly the Tsedey variety. The commercial white variety showed the same cell structure, but less pronounced and the studied brown varieties showed a smoother seed coat. The studied varieties showed otherwise a similar morphology and similar germination pattern.

### Germination under different temperatures

**Fig. 2:**
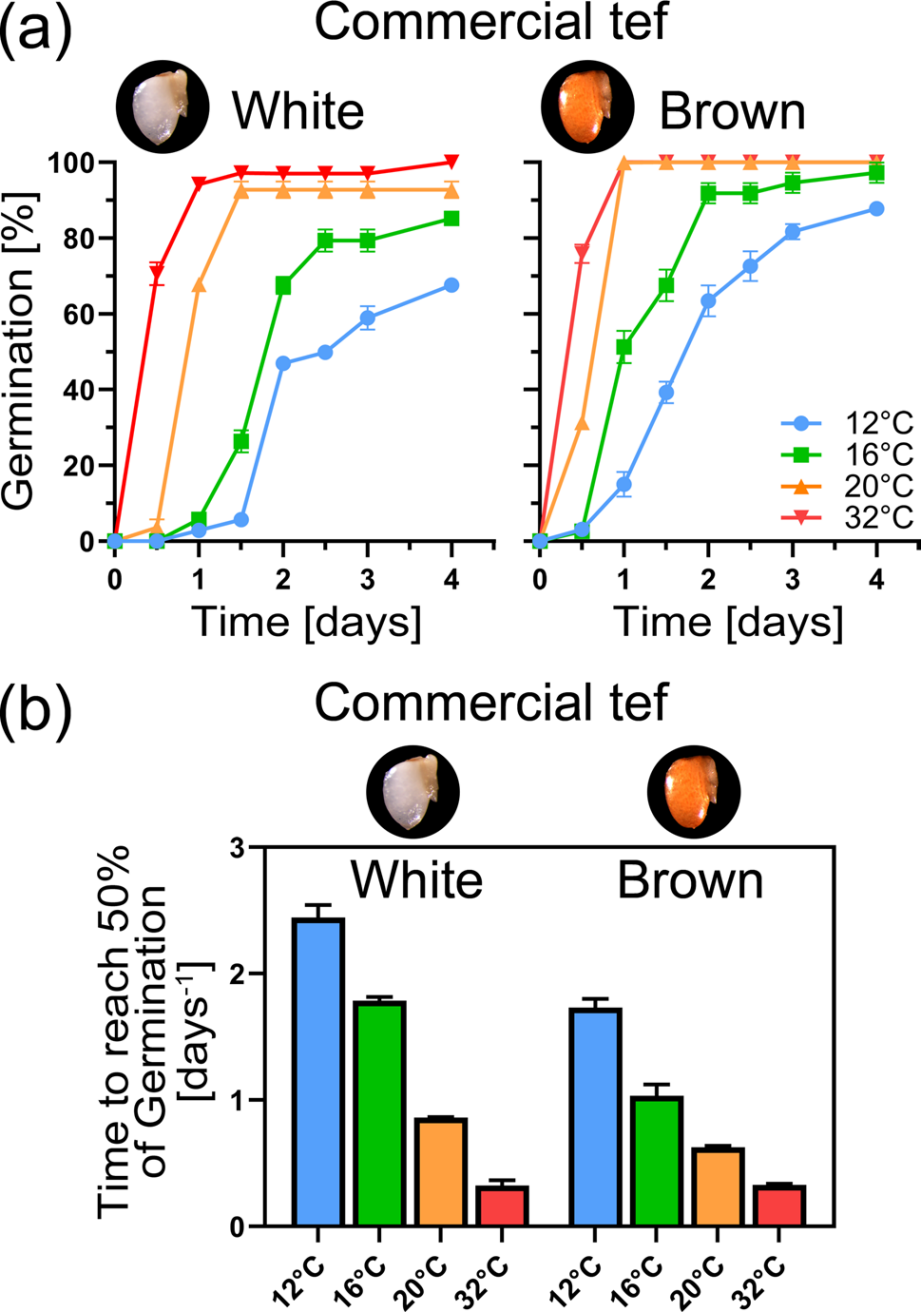
Temperature effects on tef germination. **a** Germination speed under different temperature regimes for white and brown commercial tef grains. The highest tested temperature (32 °C) lead to fastest germination (T50% = 8.5 h and 7.9 h, respectively) in both grain types, while the lowest (16 °C) showed the slowest germination speed (T50% = 60 h and 41.3 h, respectively). **b** Germination rate at 50% germination for white and brown commercial tef grains. Mean values ± SE (*n* = 3)

The germination speed of untreated white and brown tef grains is highly dependent on temperature in all studied batches (white and brown commercial batch, three batches of the white Tsedey variety, brown Swiss commercial variety). Figure 2 shows that higher temperature (32 °C) led to quicker germination speed and higher total germination percentage (G_max_) after 4 days compared to lower temperatures (12 °C, 16 °C, 20 °C). The compared white and brown grain variety showed a very similar response. Under the most optimal tested temperature, the white seeds achieved 50% germination within 8.5 h and the brown seeds within 7.9 h. Under the least optimal temperature, 50% germination was achieved after 60 h and 41.3 h, respectively.

### Priming of tef grains

**Fig. 3:**
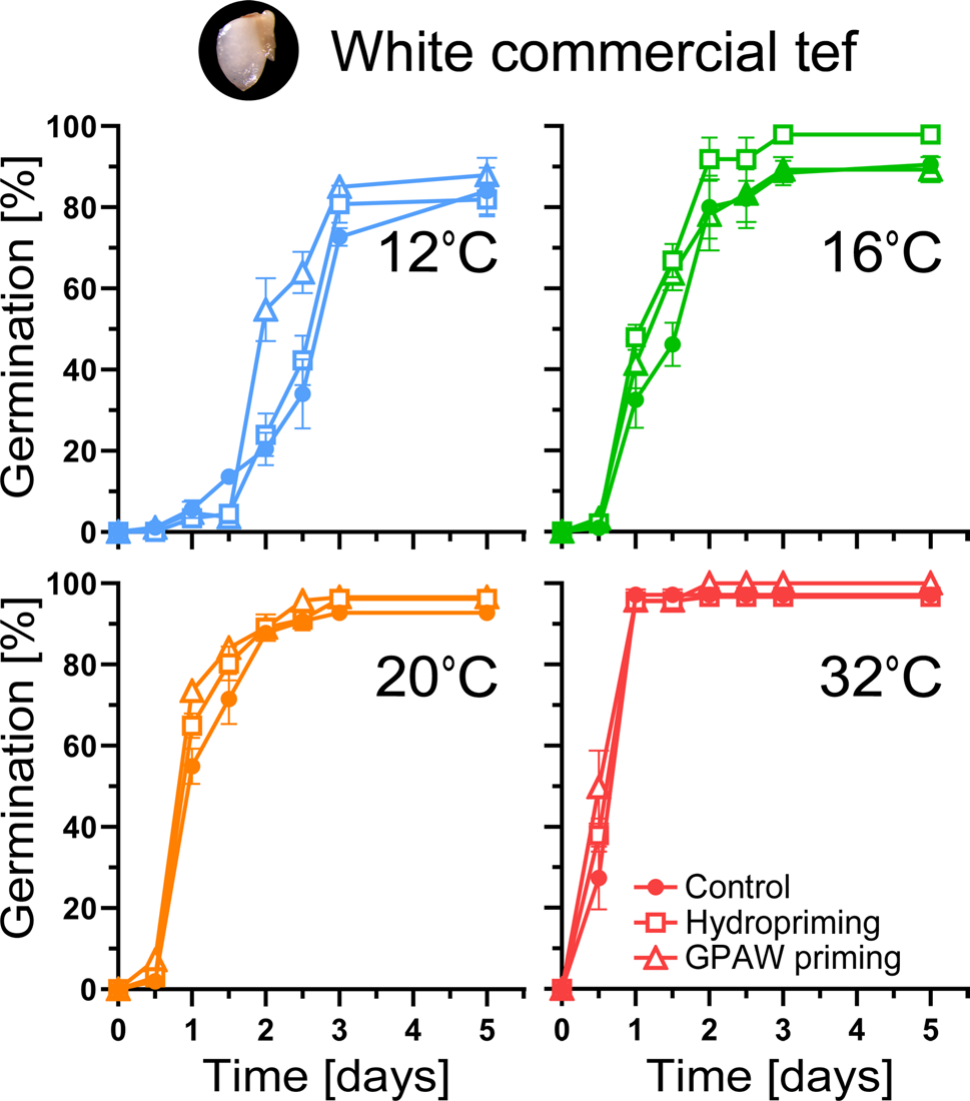
Comparison of germination speed in a commercial white tef variety for untreated, hydroprimed and gas plasma-activated water (GPAW) primed grains. The brown commercial variety showed a similar response (data not shown). Under an optimal germination temperature (32 °C), untreated and primed grains showed no significant difference in their germination speed. Favourable conditions (16 °C, 20 °C) showed slightly faster germination in primed grains compared to the untreated control. Under sub-optimal conditions (12 °C) GPAW primed grains germinated faster. Mean values ± SE (*n* = 3)

Tef grains were primed with a classical hydropriming and with GPAW. Figure 3 shows the germination speed of primed and unprimed grains under four different temperature regimes. Under sub-optimal germination conditions (12 and 16 °C) germination was quicker in primed seeds (cf. Suppl. Table S1), with GPAW primed seeds having an even greater advantage than hydroprimed seeds in 12 °C compared to the untreated control (*P* < 0.001, Suppl. Table S1). This advantage was more pronounced at lower, less optimal temperatures for tef germination, while grains at the more optimal temperatures (20 and 32 °C) show no significant difference between treated and untreated samples (Suppl. Table S1).

### Effect of accelerated ageing treatment on primed tef grains

Accelerated ageing experiments (mild 70% RH, 3/7 d, harsh 80% RH 3/7 d) have been performed on untreated, primed and GPAW primed grains. Two contrasting temperatures (12 °C and 20 °C) were chosen to monitor germination performance after the ageing treatment.

**Fig. 4:**
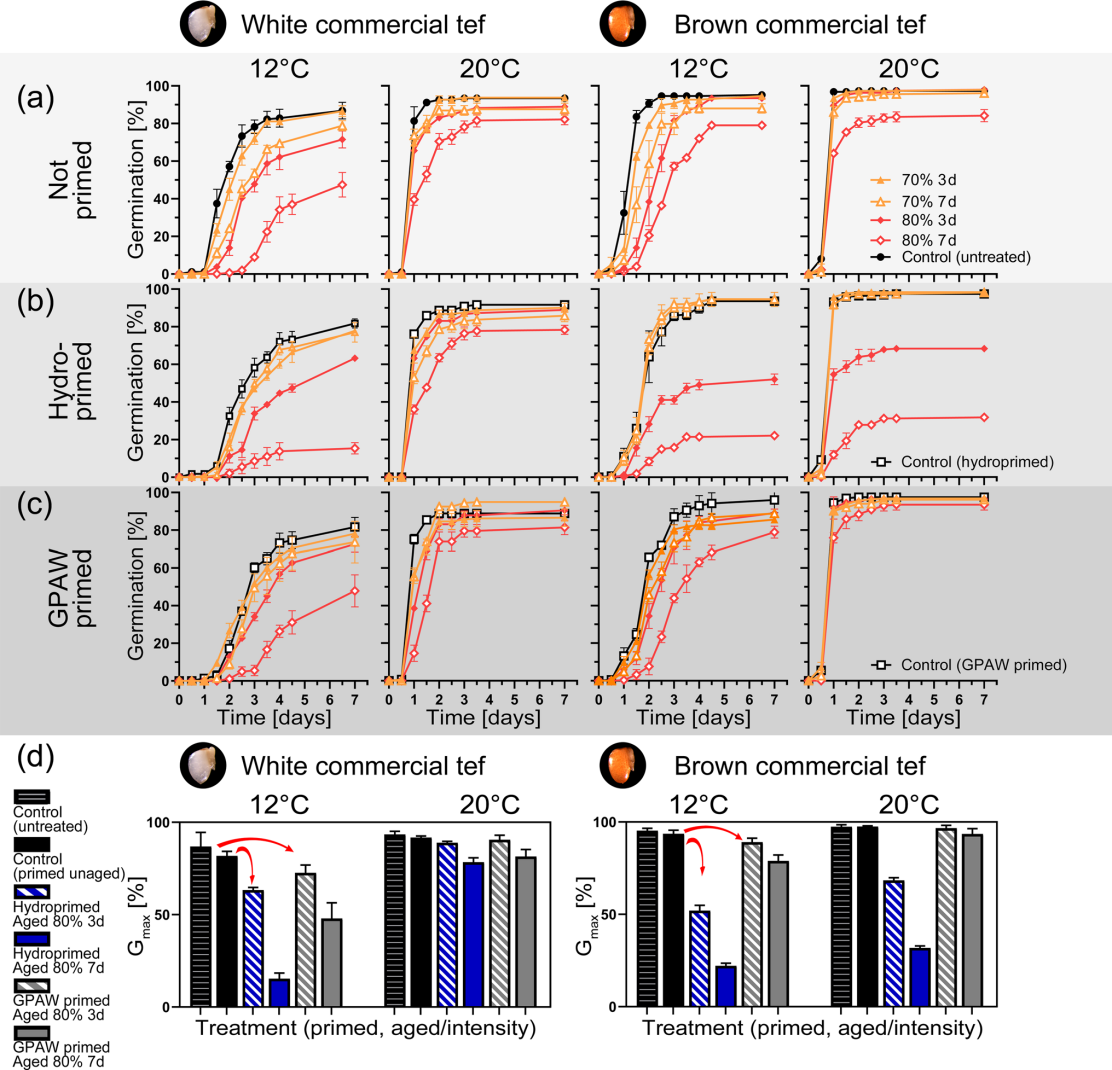
Artificial ageing of untreated and primed grains for the white and brown commercial tef variety. **a** Germination graphs (at 12 °C and 20 °C) for the different ageing intensities for the untreated grains. **b** Germination graphs (at 12 °C and 20 °C) for the different ageing intensities for the hydroprimed grains. **c** Germination graphs (at 12 °C and 20 °C) for the different ageing intensities for the GPAW primed. **d** Comparison of G_max_ for unaged primed grains versus aged (80% 3 d and 80% 7 d) hydroprimed and GPAW primed at 12 °C and 20 °C. Black lines show the control, which, in **a** are untreated seeds, and in **b** and **c** are seeds that are primed but not aged (primed controls). Coloured lines with filled markers show seeds that were aged for 3 days and coloured lines with empty markers show seeds that were aged for 7 days. Orange (70%) and red (80%) colours represent different relative humidities (RH) during ageing. Mean values ± SE (*n* = 3)

It can be observed that ageing of tef grains resulted in reduced germination speed and G_max_ in both white and brown grain varieties (Fig. 4a, Suppl. Tables S2, S3). Hydroprimed and aged seeds showed slower germination speed and lower G_max_ compared to primed and not aged seeds (Fig. 4b). The higher the relative humidity at which the seeds were aged, and the longer the ageing duration, the more pronounced the ageing effect was on seed germination. Furthermore, the germination assays at 20 °C showed a less detrimental effect of artificial ageing on tef compared to the germination assays at 12 °C. Ageing of tef grains at 80% RH for 7 days was the harshest treatment with the greatest reduction of germination speed and G_max_ compared to the control.

Compared to the hydroprimed tef grains, GPAW primed tef grains were more resistant to ageing (Fig. 4c). Particularly, GPAW primed and aged grains show an increased germination speed and significantly higher G_max_ compared to hydroprimed and aged grains under harsher ageing conditions, such as with 80% RH for a duration of 7 days (Suppl. Tables S2, S3). Germination parameters for all tested grain varieties are listed in Suppl. Table S4.

### Effect of priming and ageing on electrical conductivity

**Fig. 5:**
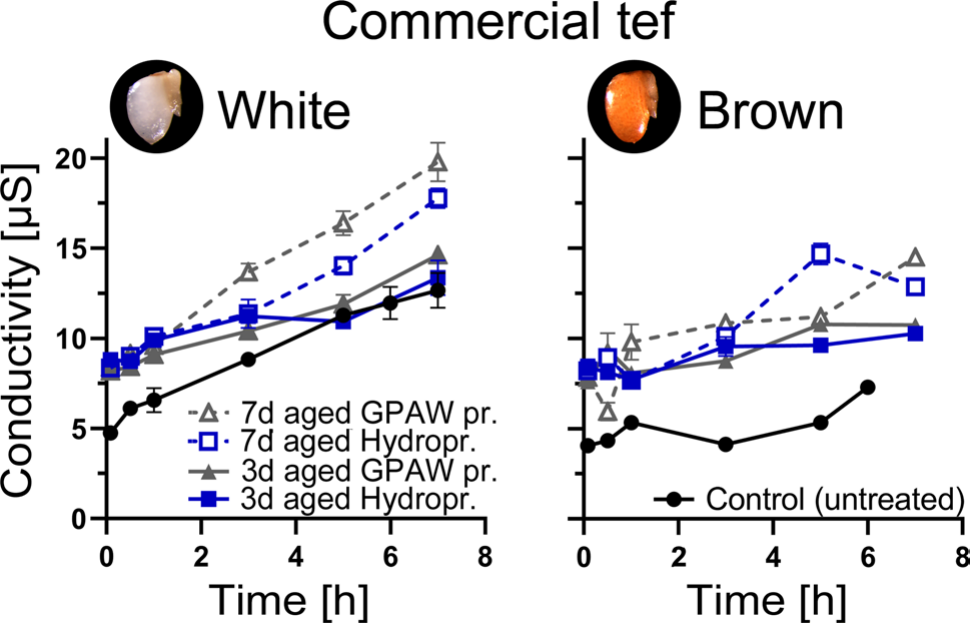
The effect of ageing on electrical conductivity (EC) of seed soak water in primed tef grains. EC of seed soak water of white and brown tef grains that were hydroprimed and GPAW primed and aged for 3 and 7 days, was compared to an untreated control. Black lines with filled round markers represent the untreated control. Blue lines represent hydropriming and grey lines GPAW priming. Filled markers show grains aged for 3 days and empty markers show grains aged for 7 days. Time [h] represents the time after incubation in 3 mL ddH_2_O. Mean values ± SE (*n* = 3) are presented

Membrane integrity, an indicator for seed vigour and sign for damage in seeds was measured by electrical conductivity (EC) of seed soak water in untreated and aged and primed white and brown grains. In Fig. 5, differences in electrical conductivity in white and brown tef, hydro and GPAW primed grains, as well as change in conductivity with regards to length of ageing treatment (3 and 7 days) are compared. Overall, white tef grains exhibited a higher EC than brown tef grains indicating that they have higher leakage. Seed soak water has potassium as a major constituent and was found to be correlated with the sugar and amino acid content (indicating general solute loss). Seed soak water of primed and aged grains showed an increased electrical conductivity compared to seed soak water of untreated grains (control) in both white and brown tef. This also increased with time, the longer the seeds were soaked in water, the higher the EC measurement of the seed soak was. Seed soak water of grains aged for 7 days had a higher electrical conductivity than those aged for 3 days, across white and brown, and hydro- and GPAW primed seeds.

### Ageing tolerance of tef grains of the Tsedey variety grown at different time-points

**Fig. 6:**
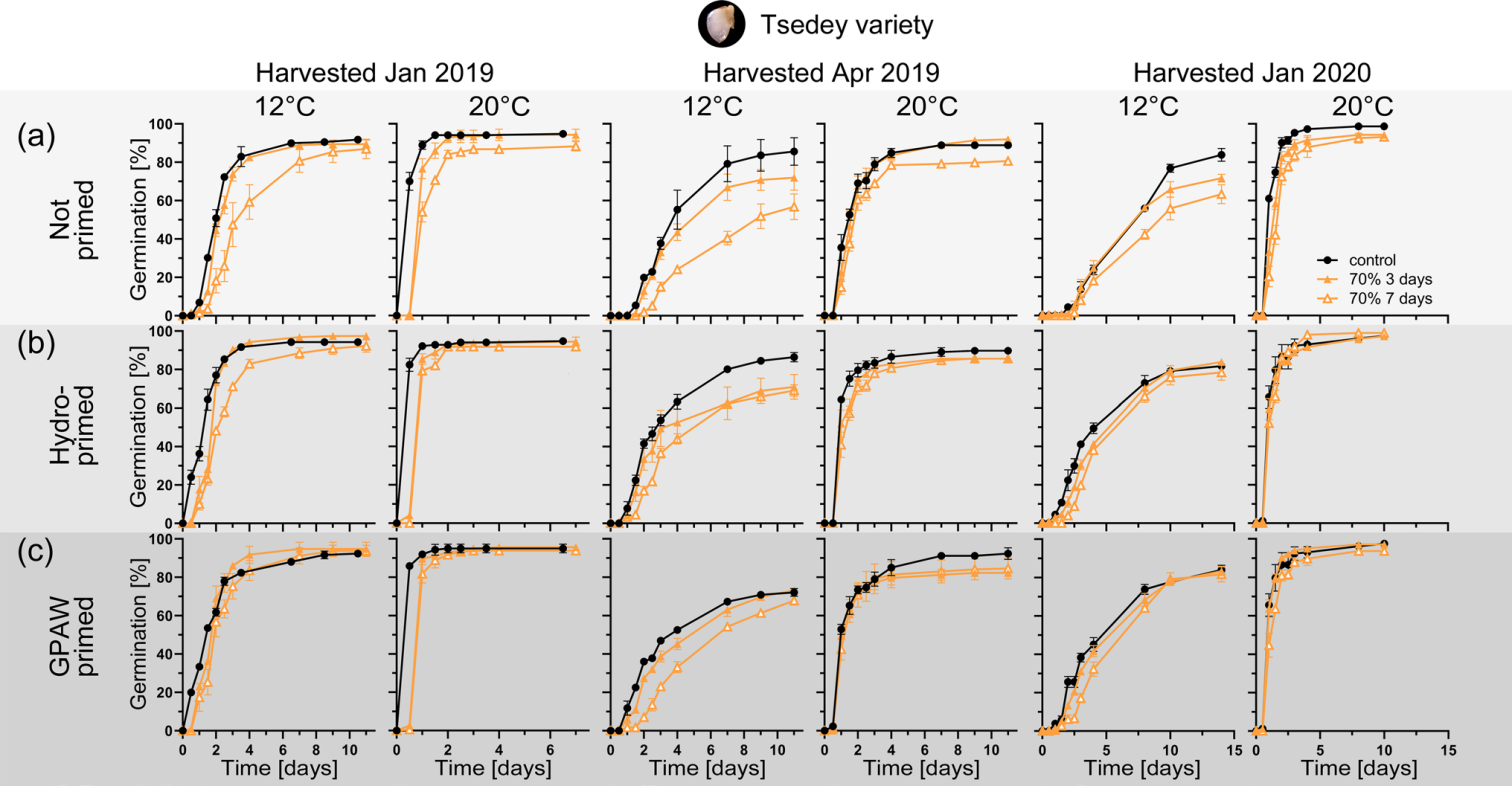
Artificial ageing of untreated and primed tef grains of the Tsedey variety. **a** Germination graphs (at 12 °C and 20 °C) for the different ageing intensities for the untreated grains. **b** Germination graphs (at 12 °C and 20 °C) for the different ageing intensities for the hydroprimed grains. **c** Germination graphs (at 12 °C and 20 °C) for the different ageing intensities for the GPAW primed grains. Tef grains were harvested January 2019, April and January 2020, respectively. Black lines show the control, which are untreated seeds and coloured lines with filled markers show seeds that were aged for 3 days and coloured lines with empty markers show seeds that were aged for 7 days at 70% relative humidity (RH) during ageing. Mean values ± SE (*n* = 3)

Three seed lots of the Tsedey variety harvested in January 2019, April 2019 and January 2020 have been examined to determine their ageing sensitivity. A screening for ageing sensitivity on the unprimed January 2020 lot revealed a sensitivity to ageing at higher moisture (80%) (Suppl. Fig. S1). All subsequent priming and ageing experiments have, therefore, been conducted at 3 days and 7 days ageing at 70% moisture content (Fig. 6). Interestingly, germination differs between grains (grown in a glasshouse in Bern) from harvests in different years and/or month with grains harvested in January 2019 showing the fastest germination. Grains grown in different years and harvested at different time-points also show different susceptibility to ageing. Tsedey harvested in January 2019 readily germinated (even under 12 °C) and only showed a slight reduction in germination speed in 70% RH 7 days ageing. Seeds harvested in April 2019 and January 2020 germinated much slower (particularly in 12 °C). Priming enhanced the germination speed while the different priming methods did not show a significant difference under the chosen ageing parameters.

### Seedling emergence of primed tef grains in pot and field experiments

**Fig. 7:**
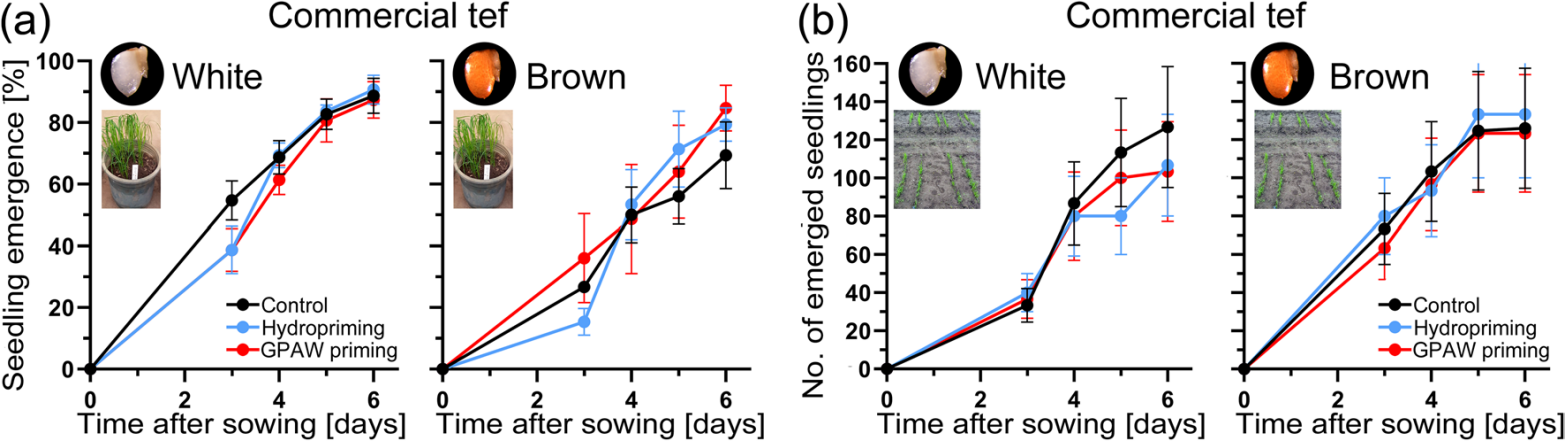
Seedling emergence of hydroprimed, GPAW primed and untreated tef grains. **a** Percentage of emerged seedling in pots (three replicates of 50 seeds) each. Average values ± SE (*n* = 3) are plotted. **b** Number of emerged seedlings in the field experiment (0.4 g of seed for 1 m^2^ hand drilled in rows)

The emergence of hydroprimed, GPAW primed and untreated tef seedlings was monitored in pots and in field experiments (Fig. 7). In pot experiments, neither experimental group showed a significant difference between treatments of varieties (brown, white). A similar pattern was observed in the field experiment. Plants did not significantly differ in their height, peduncle length or shoot biomass (Suppl. Table S5).

## Discussion

Priming is an easy to apply, low-cost widely used seed technology that leads to improved seed performance (such as increased germination rate and germination in a broader temperature range, improved seedling vigour and improved stress tolerance) (Paparella et al. 2015). Seed vigour is defined as ‘the sum of those properties that determine the activity and performance of seed lots of acceptable germination in a wide range of environments’ (ISTA 2015). Primed tef grains showed increased seed performance, particularly under less optimal conditions (12 °C) (Fig. 3, Suppl. Table S1). All tested varieties showed a similar response and both types of priming technologies used (hydropriming, GPAW priming) showed an increase in germination speed (vigour). None of the priming treatments had a negative effect on the plant health or its performance in the field. If a seed lot loses its vigour, it loses its physiological functions that allow them to perform, a process called ageing (or deterioration).

Particularly in crop seeds, increased seed and seedling performance will enhance food security. An earlier, more uniform crop emergence is associated with higher growth rates and higher yield and crop quality under stresses (Farooq et al. 2019). Furthermore, an increased seedling performance and higher growth rates could lead to advantage in weed competitiveness. For tef, weed competition has a major effect on the yield and the economic return (Gebrehiwot et al. 2020). Although seed priming has undoubtedly proven to be a valuable tool in seed science and agriculture, it has also been shown to have a negative impact on seed longevity (Fabrissin et al. 2021). Variation in the storage tolerance of seeds can be caused by differences in genotypes, growth conditions or by seed treatments such as priming. The deterioration can be tested by a combination of high moisture and temperature levels or storage under high-pressure oxygen conditions (Hourston et al. 2020). Primed seeds are often more prone to seed ageing and results in a reduced shelf-life, which has previously been demonstrated in accelerated seed ageing experiments for a variety of seeds (for example, *Lactuca sativa* L., *Brassica oleracea*, *Capsicum annuum*, *Helianthus annuus* L.) (Chojnowski et al. 1997; Buitink et al. 2000; Soeda et al. 2005; Schwember and Bradford 2010). However, it has also been shown that priming can have variable effects on subsequent survival during experimental storage (improved or decreased survival rates) (Butler et al. 2009; Fabrissin et al. 2021). Seed storage is crucial to seed producers, farmers, restoration practitioners and end users as it is vital for maintaining seed viability. Seed ageing causes the metabolic system to slow down and can result in a reduced vigour as the seed germination is slowed down (Walters et al. 2010; Colville and Pritchard 2019). The studied tef grains showed a reduction in longevity in response to high humidity and high temperature treatment. This reduction in vigour and viability was increased in primed grains and particularly in hydroprimed grains. In contrast to hydropriming, GPAW priming maintained germination particularly in more optimal temperature regimes for the white and the brown varieties. A reduced seed vigour and reduced seed viability due to ageing have a tremendous effect on germplasm preservation as well as agricultural production. A viable seed is capable of germination under suitable conditions. Reducing the negative effects on seed priming by fine tuning various priming parameters (such as imbibition, temperature and storage) as well as further research into the mechanisms of ageing and novel technologies (such as GPAW priming) have the potential to boost seed viability and have potential to increase food security.

"Plasma agriculture" is a rapidly expanding field in which pre- and post-harvest applications provide innovative agricultural solutions aiding the sustainable production of food (Bourke et al. 2018; Ito et al. 2018). Seed treatments with GPAW as well as plasma treatment of dry seeds have resulted in enhanced germination performance (Bormashenko et al. 2015; Zhou et al. 2016; Bafoil et al. 2018). Recently, Grainge et al. (2022b) revealed the underlying molecular mechanisms by which GPAW releases physiological dormancy of *Arabidopsis thaliana* seeds; in particular, how GPAW interacts with signalling pathways targeting gibberellin and abscisic acid metabolism and the expression of downstream cell wall remodelling genes. The GPAW triggered dormancy release by synergistic interaction between plasma-generated reactive chemical species and gene expression as well as direct chemical action of GPAW on cell walls resulted in premature endosperm weakening. Weakening of the endosperm and removing coat dormancies is an important process during seed germination (Steinbrecher and Leubner-Metzger 2017). GPAW, therefore, removes seed dormancy blocks by triggering multiple molecular signalling pathways combined with direct chemical tissue weakening to permit seed germination. Grainge et al. (2022b) concluded that GPAW improved seed quality by mimicking permissive environments in which sensing and integration of multiple signals lead to dormancy release and germination.

To further investigate the seed ageing in primed and untreated seeds, we employed an electrical conductivity (EC) vigour assay to compare leakage of solutes from seeds during imbibition. The EC test is one of only two vigour tests included in the ISTA Rules for Seed Testing for garden peas (*Pisum sativum*) (Matthews and Powell 2006). It has previously been shown that seed ageing or seed storage under high temperature conditions lead to increased leakages of solutes from seeds (increased electrical conductivity EC). Examples include rocket (*Eruca sativa*) seeds (Chandler et al. 2020), crop seeds (*Zea mays* L*.*) (Fessel et al. 2006) and wild species (Marin et al. 2018), but the EC has also been shown to be unreliable as a vigour test for some species (Argerich and Bradford 1989). An increased susceptibility of aged seeds to imbibition damage is seen to be caused by the weakening of cell membranes by physiological deterioration, resulting in membranes that are more sensitive to physical damage during imbibition (Matthews and Powell 2006). All tested tef grains showed higher leakage after priming and ageing treatment. The white commercial variety showed a higher EC compared to the brown variety. The brown tef showed higher sensitivity to ageing although it showed less leakage of solutes and the improvement in storability due to GPAW priming was not linked to EC as both priming treatments resulted in an equally high leakage. The beneficial effects of the GPAW priming treatment are, therefore, unlikely to be linked to membrane integrity alone.

The environmental conditions during seed production play a vital role on the properties of progeny seeds (Penfield and MacGregor 2017). Temperature has been identified as a strong signal and even very small differences have been shown to influence seed dormancy (Springthorpe and Penfield 2015). In species with coat-imposed dormancy, the seed and fruit coat properties (e.g. thickness, mechanical properties, morphological properties) are a decisive component of this trait (Dorne 1981; Sperber et al. 2017; Francoz et al. 2018; Fernández et al. 2021; Nakabayashi and Leubner-Metzger 2021). Seed longevity is a complex seed quality trait that is acquired on the mother plant during seed development and strongly influenced by the environment (Zinsmeister et al. 2020). The genome and the transcriptome of the tef genotype Tsedey (DZ-Cr-37) were sequenced by the Tef Improvement Project (Cannarozzi et al. 2014) and has been shown to possess salt tolerance. We propose that the maternal environment for the examined Tsedey variety caused the contrasting germination performance and ageing tolerance observed in Fig. 6 and Supplementary Fig. S1. Tef growth under different environmental conditions such as temperature, drought and waterlogging results in distinct plant and grain morphology, including grain mass and amount of surface mucilage (Kreitschitz et al. 2009; Cannarozzi et al. 2018; Paff and Asseng 2018). Such “genotype × environment” interactions are also known from other species where they affect key traits such as seed dormancy, seed longevity and the timing of seedling emergence (Lee et al. 2019; Farnocchia et al. 2021; Dong et al. 2022). Further research is needed to investigate the mechanisms of these “genotype × environment” interactions on tef grain morphology and physiology.

Tef represents an ideal study crop for advances in seed technologies as it is important for Ethiopian food security and a significant source of income for smallholder farmers. The growing human population, climate change and inadequate production practices threaten agricultural production practices and food security in a wide range of countries (and particularly in low-income countries) (Deressa and Hassan 2009). Eluding the negative effects of seed ageing and boosting seed quality is therefore increasingly urgent. Improved tef grain through gas plasma technology or other seed enhancements resulting in a higher stress tolerance and particularly better storage capability could contribute towards sufficient and nutritious food to work towards food security for an active and healthy life.

### Supplementary Information

Below is the link to the electronic supplementary material.

Supplementary file1 (DOCX 245 KB)

## Data Availability

All data presented or analysed in this published article are available online through figshare 10.17637/rh.25249729.v1.

## Abbreviations

EC: Electrical conductivity
GPAW: Gas plasma-activated water
MC: Moisture content
RH: Relative humidity
