# Supplementary material for: Seed priming with gas plasma-activated water in Ethiopia’s “orphan” crop tef (Eragrostis tef)
Source: Planta. 2024 Feb 26;259(4):75. doi: 10.1007/s00425-024-04359-5 (PMC10896766; doi:10.1007/s00425-024-04359-5)
Supplement: Supplementary file 1 — Supplementary file1 (DOCX 245 KB) [file 425_2024_4359_MOESM1_ESM.docx]

Seed priming with gas plasma-activated water in Ethiopia’s “orphan” crop tef (*Eragrostis tef*)

Lena Fatelnig^1^, Solomon Chanyalew^2^, Mahilet Tadesse^2^, Worku Kebede^2^, Nigusu Hussein^2^, Felipe Iza^3,4^, Zerihun Tadele^2,5^, Gerhard Leubner-Metzger^1,6^ and Tina Steinbrecher^1,^*

^1^Department of Biological Sciences, Royal Holloway University of London, Egham, Surrey, TW20 0EX, United Kingdom, Web: 'The Seed Biology Place' - www.seedbiology.eu

^2^Ethiopian Institute of Agricultural Research, Debre Zeit Agricultural Research Center, P.O. Box 32, Debre Zeit, Ethiopia

^3^Wolfson School of Mechanical, Electrical and Manufacturing Engineering, Loughborough University, Leicestershire, LE11 3TU, United Kingdom

^4^Division of Advanced Nuclear Engineering, Pohang University of Science and Technology (POSTECH), Pohang, Gyeongbuk 790-784, South Korea

^5^Institute of Plant Sciences, University of Bern, Altenbergrain 21, Bern, CH-3013, Switzerland

^6^Laboratory of Growth Regulators, Palacký University and Institute of Experimental Botany, Czech Academy of Sciences, Olomouc, Czech Republic

*Correspondence: [tina.steinbrecher@rhul.ac.uk](mailto:tina.steinbrecher@rhul.ac.uk),

ORCID ID: <https://orcid.org/0000-0003-3282-6029>

**Supplementary Table S1** Time taken for germination to reach 50% of germination (T50%) and ANOVA followed by post-hoc tests of T50% for data represented in Fig. 3. T(50%) is given as the average of the 3 biological replicates (each containing 50 grains) ± standard deviation. White commercial tef has been untreated (control), hydroprimed or GPAW primed and germinated under 12,16,20 and 32°C. *P* values from Tukey's multiple comparisons test are presented as ****, *P* < 0.0001; ***, *P* = 0.0001 to 0.001; **, *P* = 0.001 to 0.01; *, *P* = 0.01 to 0.05; ns (not significant), *P* > 0.05

| **Time to reach T(50%) in days** | **Control** | **Hydroprimed** | **GPAW primed** |
| --- | --- | --- | --- |
| **12°C** | 2.68 ± 0.12 | 2.59 ± 0.12 | 2.03 ± 0.20 |
| **16°C** | 1.54 ± 0.18 | 1.1 ± 0.10 | 1.16 ± 0.17 |
| **20°C** | 0.96 ± 0.06 | 0.88 ± 0.03 | 0.82 ± 0.01 |
| **32°C** | 0.65 ± 0.07 | 0.6 ± 0.05 | 0.51 ± 0.13 |

| **12°C** | **Control** | **Hydroprimed** | **GPAW primed** |
| --- | --- | --- | --- |
| **Hydroprimed** | ns |  |  |
| **GPAW primed** | **** | *** |  |

| **16°C** | **Control** | **Hydroprimed** | **GPAW primed** |
| --- | --- | --- | --- |
| **Hydroprimed** | ** |  |  |
| **GPAW primed** | * | ns |  |

| **20°C** | **Control** | **Hydroprimed** | **GPAW primed** |
| --- | --- | --- | --- |
| **Hydroprimed** | ns |  |  |
| **GPAW primed** | ns | ns |  |

| **32°C** | **Control** | **Hydroprimed** | **GPAW primed** |
| --- | --- | --- | --- |
| **Hydroprimed** | ns |  |  |
| **GPAW primed** | ns | ns |  |

**Supplementary Table S2** Maximum % of germination (G_max_) and ANOVA followed by post-hoc tests of G_max_ for data presented in Fig. 4 (white grains). G_max_ is given as the average of the 3 biological replicates (each containing 50 grains) ± standard deviation. White commercial tef has been untreated (control), hydroprimed or GPAW primed and aged for 3 days or 7 days under 80% RH. *P* values from Tukey's multiple comparisons test are presented as ****, *P* < 0.0001; ***, *P* = 0.0001 to 0.001; **, *P*= 0.001 to 0.01; *, *P* = 0.01 to 0.05; ns (not significant), *P* > 0.05

| **G_max_ [%]** | **Control** | | | **Hydroprimed** | | | **GPAW primed** | | |
| --- | --- | --- | --- | --- | --- | --- | --- | --- | --- |
| **White** |  | **80% RH** | |  | **80% RH** | |  | **80% RH** | |
|  | **C** | **3d** | **7d** | **C** | **3d** | **7d** | **C** | **3d** | **7d** |
| **12°C** | 86,9 ± 7.7 | 71.6 ± 7.8 | 47.4 ± 11.3 | 81.7 ± 4.2 | 63.3 ± 2.5 | 15.4 ± 5.3 | 81.7 ± 8.8 | 72.6 ± 7.3 | 47.9 ± 14.7 |
| **20°C** | 97.4 ± 1.1 | 98.1 ± 0.4 | 84.2 ± 5.7 | 97.5 ± 0.8 | 68.4 ± 2.4 | 31.8 ± 1.9 | 97.6 ± 2.3 | 96.6 ± 2.6 | 93.5 ± 5.0 |

| **12°C White** | Control | Control 80% 3d aged | Control 80% 7d aged | Hydro primed | Hydro primed 80% 3d aged | Hydro primed 80% 7d aged | GPAW | GPAW primed 80% 3d aged |
| --- | --- | --- | --- | --- | --- | --- | --- | --- |
| Control 80% 3d | ns |  |  |  |  |  |  |  |
| Control 80% 7d | *** | ns |  |  |  |  |  |  |
| Hydro primed | ns | ns | ** |  |  |  |  |  |
| Hydro primed 80% 3d | ns | ns | ns | ns |  |  |  |  |
| Hydro primed 80% 7d | **** | **** | ** | **** | **** |  |  |  |
| GPAW | ns | ns | ** | ns | ns | **** |  |  |
| GPAW primed 80% 3d | ns | ns | * | ns | ns | **** | ns |  |
| GPAW primed 80% 7d | *** | ns | ns | ** | ns | ** | ** | * |

| **20°C White** | Control | Control 80% 3d aged | Control 80% 7d aged | Hydro primed | Hydro primed 80% 3d aged | Hydro primed 80% 7d aged | GPAW | GPAW primed 80% 3d aged |
| --- | --- | --- | --- | --- | --- | --- | --- | --- |
| Control 80% 3d | ns |  |  |  |  |  |  |  |
| Control 80% 7d | * | ns |  |  |  |  |  |  |
| Hydro primed | ns | ns | ns |  |  |  |  |  |
| Hydro primed 80% 3d | ns | ns | ns | ns |  |  |  |  |
| Hydro primed 80% 7d | ** | * | ns | ** | * |  |  |  |
| GPAW | ns | ns | ns | ns | ns | * |  |  |
| GPAW primed 80% 3d | ns | ns | ns | ns | ns | * | ns |  |
| GPAW primed 80% 7d | * | ns | ns | * | ns | ns | ns | ns |

**Supplementary Table S3** Maximum % of germination (G_max_) and ANOVA followed by post-hoc tests of G_max_ for data presented in Fig. 4 (brown grains). G_max_ is given as the average of the 3 biological replicates (each containing 50 grains) ± standard deviation. Brown commercial tef has been untreated (control), hydroprimed or GPAW primed and aged for 3 days or 7 days under 80% RH. *P* values from Tukey's multiple comparisons test are presented as ****, *P* < 0.0001; ***, *P* = 0.0001 to 0.001; **, *P* = 0.001 to 0.01; *, *P* = 0.01 to 0.05; ns (not significant), *P* > 0.05

| **G_max_ [%]** | **Control** | | | **Hydroprimed** | | | **GPAW primed** | | |
| --- | --- | --- | --- | --- | --- | --- | --- | --- | --- |
| **Brown** |  | **80% RH** | |  | **80% RH** | |  | **80% RH** | |
|  | **C** | **3d** | **7d** | **C** | **3d** | **7d** | **C** | **3d** | **7d** |
| **12°C** | 95.3 ± 1.4 | 93.5 ± 1.6 | 79.0 ± 3.4 | 93.6 ± 3.5 | 52.0 ± 5.0 | 22.1 ± 2.5 | 92.9 ± 6.5 | 89.1 ± 3.6 | 78.9 ± 5.5 |
| **20°C** | 93.5 ± 1.7 | 89.0 ± 1.2 | 82.2 ± 4.9 | 91.7 ± 1.5 | 89.0 ± 1.2 | 78.4 ± 4.2 | 88.9 ± 1.5 | 90.5 ± 4.3 | 81.4 ± 6.7 |

| **12°C Brown** | Control | Control 80% 3d aged | Control 80% 7d aged | Hydro primed | Hydro primed 80% 3d aged | Hydro primed 80% 7d aged | GPAW | GPAW primed 80% 3d aged |
| --- | --- | --- | --- | --- | --- | --- | --- | --- |
| Control 80% 3d | ns |  |  |  |  |  |  |  |
| Control 80% 7d | ** | ** |  |  |  |  |  |  |
| Hydro primed | ns | ns | ** |  |  |  |  |  |
| Hydro primed 80% 3d | **** | **** | **** | **** |  |  |  |  |
| Hydro primed 80% 7d | **** | **** | **** | **** | **** |  |  |  |
| GPAW | ns | ns | * | ns | **** | **** |  |  |
| GPAW primed 80% 3d | ns | ns | ns | ns | **** | **** | ns |  |
| GPAW primed 80% 7d | ** | ** | ns | ** | **** | **** | ** | ns |

| **20°C Brown** | Control | Control 80% 3d aged | Control 80% 7d aged | Hydro primed | Hydro primed 80% 3d aged | Hydro primed 80% 7d aged | GPAW | GPAW primed 80% 3d aged |
| --- | --- | --- | --- | --- | --- | --- | --- | --- |
| Control 80% 3d | ns |  |  |  |  |  |  |  |
| Control 80% 7d | ** | *** |  |  |  |  |  |  |
| Hydro primed | ns | ns | ** |  |  |  |  |  |
| Hydro primed 80% 3d | **** | **** | *** | **** |  |  |  |  |
| Hydro primed 80% 7d | **** | **** | **** | **** | **** |  |  |  |
| GPAW | ns | ns | *** | ns | **** | **** |  |  |
| GPAW primed 80% 3d | ns | ns | ** | ns | **** | **** | ns |  |
| GPAW primed 80% 7d | ns | ns | * | ns | **** | **** | ns | ns |

**Supplementary Table S4** Germination speed of all studied tef grain varieties. Average time [days] ± SE to reach 50% of germination for primed and untreated grains. Grains have been primed with hydropriming or GPAW priming at 20% MC for 15 h and aged at 70% RH for 3 and 7 days (0 days = unaged control). Shown are the values for three Tsedey batches (harvested January 2019, April 2019 and January 2020), a brown tef batch received from a Swiss farm and two commercial tef batches (Lovegrass Ltd, Kenly, UK, white batch no 20120, brown batch no 200707)

|  |  | Not primed | | | Hydroprimed | | | GPAW primed | | |
| --- | --- | --- | --- | --- | --- | --- | --- | --- | --- | --- |
|  |  | 0 d | 3 d | 7 d | 0 d | 3 d | 7 d | 0 d | 3 d | 7 d |
| Tsedey Jan 2019 | 12°C | 2.0 ± 0.1 | 2.2  ± 0.1 | 3.5  ± 0.6 | 1.4 ± 0.1 | 1.7 ± 0.1 | 2.1 ± 0.1 | 1.4 ± 0.0 | 1.7 ± 0.1 | 2.0 ± 0.2 |
|  | 20°C | 0.4 ± 0.0 | 0.8  ± 0.0 | 1.0  ± 0.1 | 0.3 ± 0.0 | 0.8 ± 0.0 | 0.8 ± 0.0 | 0.3 ± 0.0 | 0.8 ± 0.0 | 0.8 ± 0.0 |
| Tsedey Apr 2019 | 12°C | 4.1 ± 0.6 | 5.0  ± 0.7 | 8.5  ± 1.0 | 2.7 ± 0.2 | 4.5 ± 1.6 | 4.9 ± 0.3 | 3.6 ± 0.2 | 4.8 ± 0.5 | 6.4 ± 0.1 |
|  | 20°C | 1.4 ± 0.2 | 1.6  ± 0.1 | 1.8  ± 0.1 | 0.9 ± 0.0 | 1.1 ± 0.1 | 1.3 ± 0.1 | 1.0 ± 0.0 | 1.1 ± 0.1 | 1.2 ± 0.1 |
| Tsedey Jan 2020 | 12°C | 7.3  ± 0.2 | 7.2  ± 0.3 | 9.9  ± 1.3 | 4.3 ± 0.3 | 5.2 ± 0.1 | 5.7 ± 0.1 | 4.6 ± 0.5 | 5.2 ± 0.4 | 6.2 ± 0.2 |
|  | 20°C | 1.4  ± 0.2 | 1.6  ± 0.1 | 1.8  ± 0.1 | 0.9 ± 0.0 | 1.1 ± 0.1 | 1.3 ± 0.1 | 1.0 ± 0.0 | 1.1 ± 0.1 | 1.2 ± 0.1 |
| Brown tef  (Swiss farm) | 12°C | 0.9  ± 0.0 | 1.2  ± 0.1 | 1.7  ± 0.0 | 0.8 ± 0.0 | 0.8 ± 0.0 | 1.1 ± 0.1 | 0.9 ± 0.0 | 0.9 ± 0.1 | 1.1 ± 0.1 |
|  | 20°C | 0.7  ± 0.0 | 0.7  ± 0.0 | 0.8  ± 0.0 | 0.7 ± 0.0 | 0.7 ± 0.0 | 0.7 ± 0.0 | 0.7 ± 0.0 | 0.7 ± 0.0 | 0.7 ± 0.0 |
| Commercial white | 12°C | 1.7  ± 0.1 | 2.2  ± 0.1 | 2.7  ± 0.2 | 2.7 ± 0.2 | 3.2 ± 0.1 | 3.1 ± 0.1 | 2.8 ± 0.0 | 2.9 ± 0.0 | 3.4 ± 0.4 |
|  | 20°C | 0.8  ± 0.0 | 0.9  ± 0.0 | 0.8  ± 0.0 | 0.8 ± 0.0 | 0.9 ± 0.1 | 1.0 ± 0.1 | 0.8 ± 0.0 | 0.9 ± 0.0 | 1.0 ± 0.0 |
| Commercial brown | 12°C | 1.1  ± 0.1 | 1.4  ± 0.0 | 1.8 ± 0.2 | 1.9 ± 0.2 | 1.8 ± 0.1 | 1.8 ± 0.0 | 1.8 ± 0.0 | 1.9 ± 0.0 | 2.2 ± 0.2 |
|  | 20°C | 0.7  ± 0.0 | 0.8  ± 0.0 | 0.8 ± 0.0 | 0.7 ± 0.0 | 0.8 ± 0.0 | 0.8 ± 0.0 | 0.8 ± 0.0 | 0.8 ± 0.0 | 0.8 ± 0.0 |


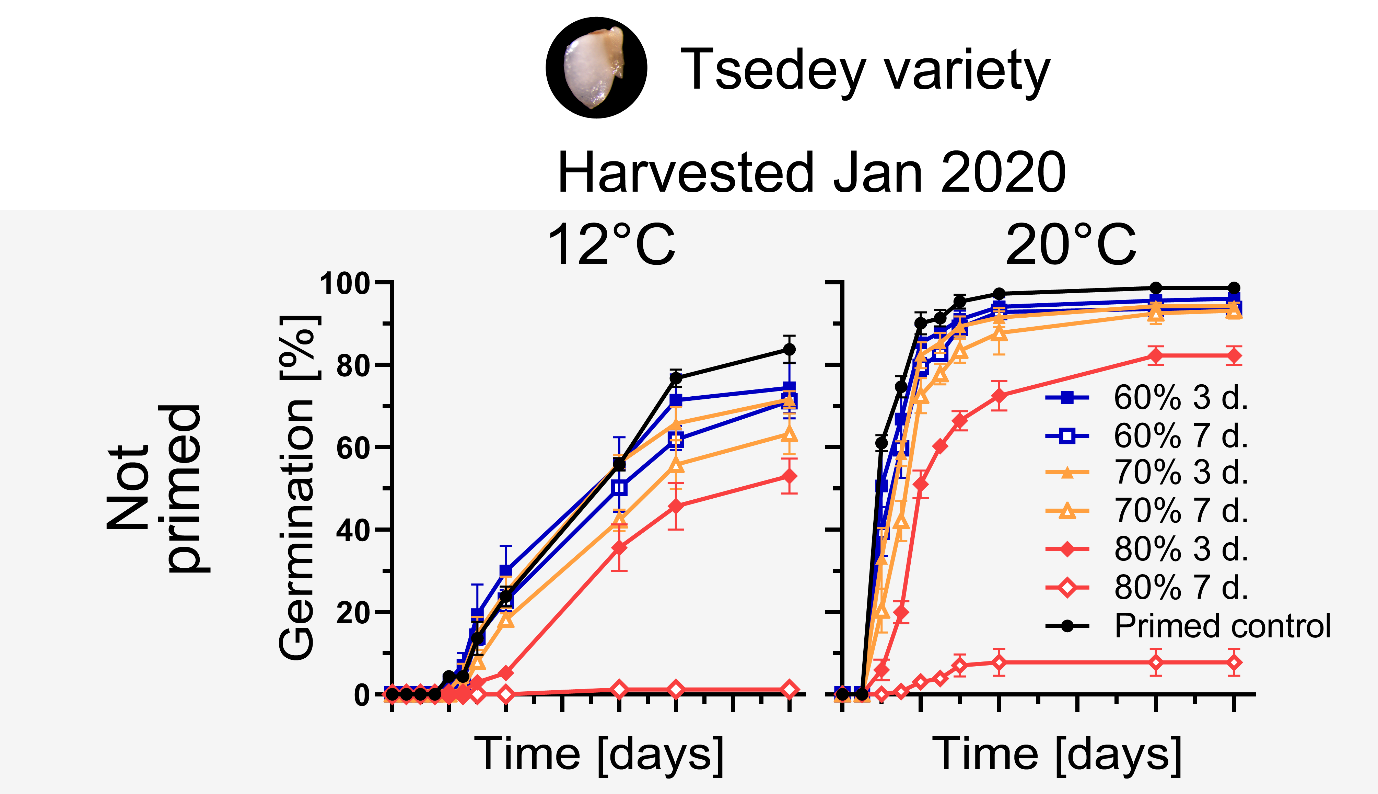


**Supplementary Fig. S1** Artificial ageing of untreated grains of the Tsedey variety harvested in January 2020. Black lines show the control, which are untreated seeds and coloured lines show seeds that were aged at 60%, 70% or 80% for 3 (filled markers) days or 7 days (empty markers), respectively. Seeds were germinated after the ageing treatment at either 12°C or 20°C. Mean values ±SE (*n* = 3)

**Supplementary Table S5** Yield-related traits for white and brown tef grains (Lovegrass Ltd, Kenly, UK, white batch no 20120, brown batch no 200707) in field and pot experiments. Grains have been either untreated, hydroprimed (HP) or GPAW primed (GPP). Mean values ±SE (*n* = 2)

|  | | **Commercial tef (white)** | | | **Commercial tef (brown)** | | |
| --- | --- | --- | --- | --- | --- | --- | --- |
|  |  | **Control** | **HP** | **GPP** | **Control** | **HP** | **GPP** |
| **Pot** | Fresh weight [mg]  / 10 plants | 14.1 | 12 | 14.5 | 12 | 12.9 | 12.3 |
|  | Dry weight [mg]  / 10 plants | 2.4 | 2.2 | 2.6 | 2.1 | 2.5 | 2.4 |
| **Field** | Plant height [cm] | 90.2  ± 7.4 | 96.6  ± 0.2 | 93  ± 8 | 91.2  ± 1.8 | 90.8  ± 4.6 | 103.3  ± 7.1 |
|  | Peduncle length [cm] | 37.2  ± 4.4 | 41.7  ± 0.5 | 39.4  ± 4 | 38.3  ± 0.7 | 38.1  ± 3.9 | 43  ± 2.4 |
|  | Shoot biomass [g] /plot | 360  ± 10 | 375  ± 25 | 350  ± 50 | 275 ±  25 | 300  ± 50 | 285  ± 35 |
